# The Coordination of Environmental Protection and Female Discrimination Based on the Concept of Affirmative Action

**DOI:** 10.3390/ijerph20043419

**Published:** 2023-02-15

**Authors:** Xia Ling

**Affiliations:** School of Law, Southeast University, Nanjing 211189, China; 230159570@seu.edu.cn

**Keywords:** affirmative action, environmental protection, women, sex discrimination

## Abstract

With the development of society, the chemical industry is expanding, and the hazy weather everywhere is becoming increasingly frequent, already affecting people’s lives and causing them to pay more attention to environmental issues. Therefore, this paper highlights the role of women in environmental protection by studying the coordination of environmental protection and female discrimination based on the concept of affirmative action. Through this study and a survey, we found that China has not yet realized that women’s participation in environmental protection plays a key role in improving the quality of our environment and the development of ecological civilization. However, we should clearly understand that environmental issues are not only personal, they are related to the survival and development of a country, and as a member of that country, both women and men should have the right and obligation to protect the environment. Therefore, this article discusses the concept and meaning of affirmative action and gender discrimination in the context of research on these concepts, discussing the problems and phenomena that women encounter in environmental protection. These include the system of women’s environmental protection, gender issues for women in society, and the unequal treatment from the Government based on some studies. Through the study and analysis of the system of women’s environmental protection, the role and position of women in this regard is summarized. Finally, it is suggested that, for the construction and development of ecological civilization in China, it is necessary to fully integrate ecological civilization into all aspects of society and pay attention to environmental protection issues. Therefore, we should pay attention to the role of women in environmental protection, provide corresponding policies, and actively encourage women to partake in environmental protection in order to build an environmentally friendly and resource-saving society together.

## 1. Introduction

Nowadays, women are working in a wider range of jobs and their knowledge and education levels are increasing. Many female workers also advocate for saving environmental resources, promoting the development of environmental protection, contributing their share to the construction of ecological civilization, and setting a good example for people around them [1]. Studies have shown that women play a key role in everyday environmental problems. The concept of “green consumption” first appeared in Western countries. Green consumption means that people should have an awareness of environmental protection in their daily life, work and study, and consider whether their own behavior has advantages and disadvantages in the development of the environment or ecological civilization. Women play an irreplaceable role in the formation and spread of this behavior. On the one hand, in most cases in daily life, it is women who buy daily necessities, and a factor that affects the environment is whether women can choose low-carbon and pollution-free products when they buy daily necessities. Therefore, women play an especially important role in this aspect. On the other hand, women also play the role of mother in the family, and in this role, words and deeds will affect the growth and development of children. Only by making efforts to improve their environmental awareness and ecological consumption concept, can women improve their awareness of protecting the environment in the process of educating their children, so that they can establish the idea of harmonious coexistence between human and nature from an early age. In future production and development, children will not wish to damage the environment due to disinterest [2,3,4,5,6,7]. The focus of environmental protection is not how to protect, but how to prevent, so the awareness of environmental protection should start from a young age, an aspect in which women play a key role.

As seen in its historical development, the progress and development of China cannot be separated from the struggle of women, and it is inseparable from the progress and development of women [8]. The top-level design can only be implemented in combination with the specific practice, and the value of the top-level design is reflected. The realization of the concept of sustainable development also requires the joint efforts of all people in all fields. A sustainable development model is worth promoting since it has a low energy consumption, low pollution and appropriate consumption. Women, as core figures in family life, can effectively practice this model. In environmental protection, women have multiple objectives, because a woman is not just a mother, but also a person, so she has a responsibility and obligation to promote sustainable development in China. In developing countries, women are the main force of promoting agricultural production. Most food is produced by women, providing the means for human survival and development. Moreover, agricultural production also has a great impact on the environment that depends on women’s cognition and consciousness in environmental protection. Therefore, we can understand that, in environmental protection activities, women have also made a great contribution to economic development. The combination of environmental protection and economic development can promote the sustainable development of China [9,10,11]. In summary, as family caregivers, children’s educators, agricultural producers and consumers in daily life, women bear great responsibilities within the concept of national sustainable development and make great contributions to China’s sustainable development.

In summary, the protection of women’s human rights is essential for the future development of China, and we must now focus on how to respect and protect human rights and promoting the implementation of affirmative action. According to the survey, women are not treated equally in environmental protection, nor do they give full play to their initiative. Most people still believe that women only need to take care of their own families, and they are not responsible for environmental protection. There is still some gender discrimination. In addition, the environmental protection system does not fully guarantee the interests of women in environmental protection. The concept of affirmative action is the key to solving ecological problems in China, which requires people to abandon the incorrect concept of machismo, redefine ecological values, and encourage women to pay attention to ecological and environmental problems. The implementation of affirmative action is conducive to enhancing the social subjectivity, participation in social activities, and abilities of women, more effectively solving the problem of environmental protection. Therefore, this paper analyzes the problem of gender discrimination in environmental protection and the implementation of the affirmative action in China with reference to relevant studies and puts forward corresponding suggestions for solving the problems encountered in this study. This provides reference values for the future practice of the affirmative action concept in China.

## 2. The Concept of Affirmative Action and Gender Discrimination

### 2.1. The Concept of Affirmative Action

Equal rights means equality between men and women, and equality between men and women is also called gender equality. This means that men and women are equal in rights, opportunities and responsibilities, and that men and women are equal in dignity and worth [12]. The concept of “equality between men and women” was first defined in the Mexican Declaration, which was put forward and adopted at the First Conference on Women’s Issues. At the Fourth Conference on Women’s Issues, the concept of equality was first combined with the issue of human rights. It was stated by the Beijing Declaration and Platform for Action that “gender equality is a human rights issue and a condition for social justice”. The definition and expression of the concept of affirmative action in these documents have a great impact on the promotion of gender equality and the development of women’s human rights in various countries [13,14,15]. On the surface, the concept of equal rights is a relatively neutral concept: men and women enjoy their rights equally, and they both give full play to their own intelligence and enjoy the fruits of participating in the political, economic and cultural development of their country. However, in essence, the concept of affirmative action actually refers to the realization of women’s human rights. This is because since ancient times, women have suffered from unequal treatment in education, politics and society, as well as serious discrimination, and robbed of their rights to equal treatment. Even now, women’s power and status are not equal to those of men. The document adopted at the Third Conference on Women’s Issues stated that “equality is not only about equal treatment of women in the law, not only about eliminating the discrimination against women in the law, but also about equal treatment of women in the opportunities and rights to participate in development”. Therefore, the fundamental goal of the concept of affirmative action is to fully realize women’s rights [16].

### 2.2. Gender Discrimination

Gender discrimination is the product of gender inequality, and the root cause of gender discrimination is that women are not treated equally [17]. In the context of historical development, the issue of combating gender discrimination has officially opened a new chapter with the adoption of the Universal Declaration of Human Rights, which states for the first time that all human beings are born free and equal in dignity and rights. In response to this principle, the United Nations has adopted many documents to proclaim this declaration, the most important of which is the Convention on the Elimination of All Forms of Discrimination against Women. For the first time in international law, the document defines “discrimination against women” as “any distinction, exclusion or restriction on the basis of sex which has the effect or purpose of preventing or depriving married or unmarried women of the enjoyment or exercise of their civil rights or any other aspect of their human rights and fundamental freedoms on the basis of equality between men and women”. In this definition, the purpose of gender discrimination is to deprive women of their freedom and rights. In the Fourth Conference on Women’s Issues, an emphasis was once again placed on eliminating any discrimination against women and girls, stressing that women’s issues should be paid attention to since the condition of human rights issues is not only to improve the status of women, but also to achieve gender equality. If women cannot enjoy equal rights in real-life situations, the human rights issues cannot be completely resolved. Human rights is a one-sided issue and cannot be treated equally in terms of gender [18,19,20,21,22,23]. These international human rights documents clearly define the relationship between gender equality and human rights, explain how to improve gender discrimination, and provide standards for the international community to recognize women’s human rights and eliminate gender discrimination.

Although there are some physical differences between men and women, there is essentially no difference regarding IQ [24]. Therefore, we should safeguard the personality and dignity of women, and vigorously practice the principle of gender equality in environmental protection. The main factor affecting gender discrimination is the ideology of male supremacy and unfair systems in society; therefore, we should fundamentally eliminate gender discrimination, building a harmonious coexistence with equality and freedom of social relations and a reasonable gender distribution of workers. This will ensure that every citizen can independently develop, consciously attach importance to environmental protection, and make their own contribution to the realization of sustainable social development [25,26,27].

## 3. Gender Discrimination in Environmental Protection

### 3.1. Women’s Environmental Interests Are not Protected

Due to the influence of traditional male power, men in our country are still in a strong position in terms of their rights and the possession of social resources: political, economic, cultural, environmental, etc. They can use their position to block or suppress the expression and eventual realization of women’s environmental interests in public participation in environmental protection activities [28]. Gender-neutral environmental and sustainable development policies tend to ignore the differences in the impacts of environmental and natural resource degradation and climate change on women and men. Some modern policies lack gender sensitivity in their formulation, and some evaluation systems lack a gender perspective for evaluating the impact of the environment on people’s survival and development, and thus there are differences in evaluating the different impacts of the environment on men and women. Gender perspectives are also lacking in some evaluation systems when assessing the impact of the environment on people’s survival and development. In addition, factors that affect the validity of gender assessments are the lack of research on women and the environment and the lack of clarity in the collection and analysis of relevant data on gender issues [29,30,31]. Therefore, according to the gender comparison, women do not have absolute equality in the expression and realization of their interests in environmental protection, and this situation should be changed by legal means.

### 3.2. Gender Equality Is not Guaranteed in the Environmental Protection System

Women’s rights in environmental protection have not been fully addressed, and thus the power of influence is limited. Women’s awareness of environmental protection needs to be further strengthened and improved, especially for those living in rural areas [32]. Human beings usually do not pay attention to the importance of gender equality in environmental protection problems, so it is difficult to realize the concept of affirmative action in environmental protection, and this cannot be achieved overnight. The number of women in our population is very large, and thus the impact on environmental protection issues is also huge. However, this influence needs to be guided. We must not hold the old concepts and traditions that men are superior to women, are in charge on the outside, and women are in charge of the inside. We should avoid discrimination against and the suppression of women, instead actively guiding and enhancing women’s awareness of environmental protection, and paying attention to women’s role in environmental protection. With the wrong attitudes, women’s environmental protection rights will be limited and deprived, and the scope of public participation in environmental protection system cannot be guaranteed. In addition, if most women have no awareness of environmental protection and have a negative attitude towards environmental issues, it is very likely to cause environmental damage and pollution [33,34,35,36,37,38,39]. Therefore, we should combine the actual situation, refer to relevant policies and experience, strengthen gender awareness in environmental protection, construct the policy of gender equality in environmental protection, and gradually improve the implementation of the concept of gender equality in environmental protection.

### 3.3. Gender Roles Assigned to Women by Society

Women all over the world have always played an important role in environmental protection issues, but mainstream theoretical research has not investigated the reasons for this. One of the reasons that cannot be ignored is that the traditional political theory of China has always disparaged the status of women and excluded women from the scope of “science” and “culture” [40]. Since the beginning of feudal society, there has always been a belief that men are superior to women: men can participate in the administration of state affairs, but women can only preside over domestic affairs at home and unconditionally obey men. In traditional society, women are assigned the roles of mother, wife and daughter-in-law. It is because of these roles that women tend to focus on family life. To some extent, this reduces the motivation of women to participate in environmental protection and hinders their motivation to contact the outside world. The moral norms of Chinese traditional culture are “male owner outside female owner inside”; therefore, women were gradually ignored by people. It was not until the 1911 Revolution that society became more conscious of women’s rights and a large number of newspapers began to promote gender equality [41,42,43,44,45].

### 3.4. The Dual Constraints of Politics and Economy

The main factor that influences women’s participation in environmental protection advocacy is the government agency, which empowers women to participate in environmental protection advocacy [46]. The media are the main source of information dissemination and environmental communication. As part of the media-influenced public, women are an important force in environmental protection. At the same time as the continuous improvement of female environmental awareness in China, governments at all levels are also implementing consultative democratic systems and strengthening the supervision of the ecological environment. In particular, new media are also vigorously promoting green concepts, but it is still very difficult for women to participate in environmental protection. On the one hand, the Government has absolute authority to speak on the topic of national political life. In addition, most women in China do not consider themselves masters of managing society. On the contrary, they trust the Government in many ways. This leads to a top–down relationship between the Government and women to rule and be ruled. Although this situation is conducive to maintaining social harmony and stability, women’s lack of motivation will likely lead to government restrictions on women’s participation in environmental protection [47,48,49,50,51]. On the other hand, the media must also to take responsibility for their own profits and losses and consider how to improve their economic profits. In this way, the media come under both political and economic pressure, which is particularly evident in the case of environmental protection.

## 4. Measures to Safeguard Women’s Rights in Environmental Protection

### 4.1. Establish a Legal Mechanism to Promote Women’s Participation in Environmental Protection

The Government has a legal obligation to promote women’s participation in environmental protection through various channels, such as receiving environmental education, holding marches and rallies. It supervises the environmental legislation and decision-making actions of governments at all levels and relevant departments, supervises the management activities of the competent administrative departments of environmental protection, and formulates the methods and procedures for the implementation of these activities. Article 7 of the Disambiguation Convention stipulates that State parties should implement appropriate measures to ensure that women participate on equal terms with men in some environmental organizations concerned with political matters [52,53,54,55]. Generally speaking, the number of Chinese women participating in environmental protection is small, its development is slow, bureaucracy is heavy, and self-construction is not perfect. With a focus on these problems, it is necessary to support women’s participation in environmental protection by means of legislation and formulation of policies, make their legal status clear, and establishment registration procedure, rights and obligations, etc. This policy of female participation in environmental protection in China is set to develop and strengthen, enabling women to participate in environmental protection. In addition, we can learn from the relevant provisions of state organs exercising legislative power according to laws in other countries.

### 4.2. Give Full Play to Women’s Potential and Enhance Women’s Rights

In order to give full play to women’s potential and improve women’s environmental protection rights, the most important point is to pay attention to women’s social status and improve their decision-making power and voice in environmental protection [56]. At present, Chinese women’s cognition and consciousness in the aspect of environmental protection are relatively low. In the face of the problems of ecological crisis, we must strengthen the education of environmental protection for women, promote women’s awareness of environmental protection, and enhance women’s environmental protection ability and level. Understanding and mastering the knowledge of environmental protection is the key to enhancing the awareness of environmental protection. Therefore, it is necessary to publicize environmental protection and incorporate it into everyday life, so that more women can accept and recognize environmental protection. Only by enabling women to master more environmental cognition can their awareness be enhanced [57,58,59,60,61]. Therefore, in order to enhance women’s awareness of environmental protection, it is necessary to start from the cultivation of women’s knowledge of environmental protection, use all kinds of environmental protection activities, strengthen women’s awareness of environmental protection, and on this basis, enhance women’s rights in environmental protection.

### 4.3. Improve Gender Awareness in Environmental Protection-Related Decision Making

We must encourage governments at all levels to incorporate gender awareness into their policies and formulate relevant environmental protection programs [62]. For example: (1) Education and training should be conducted in accordance with relevant policies in environmental protection in order to enhance the environmental protection awareness of decision makers, sensitize decision makers to gender issues, and enhance decision making regarding mainstream gender issues. (2) We must learn lessons from women’s approaches in policy and program development, greatly reflect the needs of women, evaluate potential gender differences, etc. In assessing the impact of the environment on human development, a gender perspective should be consciously incorporated, i.e., an effective mechanism for collecting, analyzing and using gender-specific data. We must strengthen research on environmental protection issues from a gender perspective, especially with regard to natural disasters and women’s relations, and evaluate policies and programs from the perspective of whether women enjoy equal access to natural resources [63,64,65,66,67].

Nowadays, in the construction of ecological civilization, the social awareness of gender is still lacking. When formulating relevant policies, it is quite common to formulate policies based on existing resource allocation and gender divisions of labor. Although current policy takes into account the physical differences between men and women and suggests ways to adapt to those differences, it does not aim to change the status quo of gender. In the process of practice, this policy has strengthened the traditional gender inequality model, and the consideration of social gender is conducive to the liberation of women and the construction of ecological civilization in the real sense. In the process of policy implementation, the promotion of gender equality should be combined with the protection of the environment for human development, and the concept of neutrality in policy implementation should be changed [68,69,70,71]. In summary, the prominent gender factor can promote the effective implementation of the policy and must be addressed.

### 4.4. Promoting Women’s Equal Participation in Environmental Management and Decision-Making

We must promote women’s full participation in the management of environmental resources and the decision making of environmental protection, improve women’s ability to participate in environmental protection, and promote sustainable social development [72]. The previous differences in the division of labor in gender discrimination will be reformed, so that women can receive different education in different fields, strengthen their knowledge, and enhance their management opportunities in environmental protection. The issue of women’s rights is a basic human rights issue. A women’s rights protection system should be incorporated into laws and regulations and internalized into social norms. In addition, women and the development of the environment are in need of legal protection and supervision. In the process of social development, the Chinese Government must improve the legal system according to the relevant practices, and strive to establish a fair and equitable social participation system [73,74,75]. We can make preparations in two aspects. First, we should improve legislation, clearly promote women’s rights, and formulate relevant policies in various laws and regulations to ensure that women can equally participate in environmental management decision-making. The second is to realize women’s legal rights. In environmental protection, the law enforcement capacity of environmental protection departments should be strengthened to ensure timely prevention and solutions to relevant problems, providing legal channels for safeguarding women’s rights.

## 5. Conclusions

In today’s society, there are an increasing number of ecological problems, and solving them is our primary consideration in modern times. As the old saying goes, “Women can hold up half of the sky”, modern women not only take care of their families and educate their children, but also participate in the use and management of environmental resources. However, studies have found that women are not treated equally in environmental protection, which means that we are not aware that women’s participation in environmental protection plays a key role in improving the quality of our environment and the development of ecological civilization. Therefore, the implementation of the concept of affirmative action in environmental protection in China is particularly important, and we should reflect on environmental issues from a gender perspective; integrate gender, environment and development; and pay attention to the role of women in environmental protection and development, thus solving environmental problems and women’s problems in China from a practical point of view.

## Data Availability

Not applicable.
