# Correction: Ling, X. The Coordination of Environmental Protection and Female Discrimination Based on the Concept of Affirmative Action. *Int. J. Environ. Res. Public Health* 2023, *20*, 3419

**DOI:** 10.3390/ijerph23070868

**Published:** 2026-07-03

**Authors:** Xia Ling

**Affiliations:** School of Law, Southeast University, Nanjing 211189, China; 230159570@seu.edu.cn

Yanhong Liu was removed from the author list, and the Author Contributions section was removed from the original publication [1]. The author states that the scientific conclusions are unaffected. This correction was approved by the Academic Editor. The original publication has also been updated.
